# Optimizing the diagnostic capacity for COVID-19 PCR testing for low resource and high demand settings: The development of information-dependent pooling protocol

**DOI:** 10.7189/jogh.10.020515

**Published:** 2020-12

**Authors:** Damir Vukičević, Ozren Polašek

**Affiliations:** 1 Department of Mathematics, Faculty of Science, University of Split, Split, Croatia; 2 Department of Public Health, University of Split School of Medicine, Split, Croatia

## Abstract

**Aim:**

To compare different pooling methods in an attempt to improve the COVID-19 PCR diagnostic capacities.

**Method:**

We developed a novel information-dependent pooling protocol (*indept*), based on transmission of less informative sequential pools on to the next pooling cycle to maximize savings. We then compared it to the *halving*, *generalized halving*, *splitting* and *hypercube* protocols in a simulation study, across variety of scenarios.

**Results:**

All five methods yielded various amount of test savings, which mostly depended on the virus prevalence in the population. In situations of low prevalence (up to 5%), *indept* had the best performance, requiring on average 20% of tests needed for singular testing across scenarios that were analyzed. Nevertheless, this comes at the expense of speed, with the worst-case scenario of *indept* protocol requiring up to twice the time needed to test the same number of samples in comparison to the *hypercube* protocol. In order to offset this, we developed a faster version of the protocol (*indeptSp*), which minimizes the number of terminal pools and manages to retain savings compared to other protocols, despite marginally longer processing times.

**Conclusion:**

The increasing demand for more testing globally can benefit from application of pooling, especially in resource-restrained situations of the low- and middle-income countries or situations of high testing demand. Singular testing in situations of low prevalence should be systematically discouraged.

Pooling is merging of similar biological samples of unknown diagnostic status, in order to optimise the use of laboratory resources. As such, it is of high interest in resource-restraint situations, regardless of the reagents, equipment or time. The emergence of COVID-19 has introduced such a situation globally, with huge increase in the diagnostic demand that requires optimization of laboratory workflow and entire health care [1,2].

The theoretical framework for successful pooling resides in several assumptions, and the principal one is that the pooling will not cause dilution that will devoid the mixture form ability to detect the positive sample from it. Pooling is only meaningful in situations of low prevalence, since an increase in prevalence renders it less effective [3,4]. Three main theoretical approaches for pooling exist; halving, generalized halving and splitting, which use different scheme to split samples in subsequent pools. In addition, several more approaches were developed to offset specific situations, including a double-averaging model under unknown prevalence [5], double pooling [6], or a multidimensional pooling, which assumes that a sample may enter the testing at multiple stages [7], using multiple combinations [8] and a non-adaptive approaches [9]. Recently, a novel approach was developed, based on the hypercube probing, which was validated and shown substantial savings are feasible, even in low- and middle-income countries [10].

Next important question is the pool size, which was suggested to ideally range between 4 and 10 samples [11-18]. Even larger pools were shown to be effective, with 32 [19-21] or even up to 80 pooled samples that were reported to be effective in laboratory-validated viral diagnostics [22]. Overall, regardless on the size of the pool, previous papers have reported fundamental savings, up to 89% fewer tests in situations with prevalence under 5% [23]. When the prevalence increases, pooling may still yield savings over the consecutive testing approach [24]. Finally, field-testing is a critical component of the overall assessment of pooling. Previous studies often did report substantial savings if pooling was applied [1,10,25], but some studies reported lower gains in real situations, compared to theoretical expectations [26,27].

The aim of this study was to compare the most prevalent pooling methods and to optimize the savings by developing a novel, information-dependent protocol.

## MATERIAL AND METHODS

This was a simulation study, based on computer-generated scenarios, with the principal aim of selecting the best pooling protocol available. First, we can define the pool size (*P*), as the number of initial samples (or swabs) that can be pooled into a single pool. We also define the number of aliquots that can be created from a single biological sample (denoted as *T*) and prevalence of virus in the population (*p*) as the main variables for the study. In addition, a number of initial assumptions must be satisfied (**Online Supplementary Document**). In addition, three main assumptions were initially put in place: (i) re-testing of the sample testing produces the same result, (ii) if one positive sample in the pool yields the positive result when individually tested, the pool would be positive, and (iii) if all the samples in the pool individually tested give negative results, then the pool would be negative (**Online Supplementary Document**).

First, we developed a novel information-dependent protocol (*indept*). This protocol utilizes information from all the tested pools, including negatives and previous pooling cycles, in order to maximize the gains through reduction of the number of test runs. This is done in a multi-dimensional fashion, through transmission of selected, less informative pools on to the next pooling cycle (denoted as *G*), where it is possible to optimize the process even further (Figure S4 in the **Online Supplementary Document**). This protocol can be demonstrated in a simple example, where two samples are pooled and their test result is positive. In the next cycle, we need to test the first sample, which if tested negative, the protocol does not require and more testing, as the second sample is positive. If the first sample is tested positive, then we do not know the status of the second sample. Now, instead of testing the second sample, we relocate it to the next pooling cycle, where we pool it with another similar situation, until we get a pool of negative result. It is imperative to build in the criterion that each biological specimen has a finite number of test runs that can be done from it (number of aliquots), therefore a pooling protocol must not violate this limitation in order to retain diagnostic ability for each biological sample.

We then compared three theoretical protocols, namely halving, *generalized halving* and *splitting* with *indept* (Figures S1-S3 in the **Online Supplementary Document**). This comparison was performed in the range of scenarios in which the three comparative protocols had the best yields, which was based on the initial pool size of 32 (*P* = 32). We then compared *indept* with the recently described *hypercube* protocol [10]. This comparison favoured the theoretical assumptions for the greatest yields of the *hypercube* protocol, with the initial pool size of 64 (*P* = 64). Both groups were compared across a range of prevalence, from 0.1 to 5%, and the aliquot number varying from *T* = 2 to *T* = 6. The main outcome measure in all instances was the percent of tests that were utilised (*PTU*), defined as number of tests needed to determine the status of every sample in the pool compared to singular (un-pooled) testing. Notably, savings can be defined as the inverse value of this number.

In addition, we defined the ratio of *PTU* (*rPTU*) as the ratio of *PTU* of *indept* over the *PTU* of remaining protocols, for any given prevalence and aliquot size combination. This allowed the direct comparison of savings of two protocols, where the value of 1.0 meant that the two protocols performed similarly, values lower than 1.0 denoted savings while values above 1.0 denoted greater cost of *indept* compared to the remaining four protocols, in relation to number of tests that were utilized. We also defined the times needed to complete the entire testing cycle. This calculation was based on a simulation of the number of cycles needed to complete the diagnostic process of all samples within the pool (**Online Supplementary Document**).

All the protocols were developed in C# and the source code is available upon request. Since no contact was made with any of the patient data or biological samples, no ethical approval was sought. The comparisons of results were made by *t* test, with significance set at *P* < 0.05.

## Results

All of the protocols yielded substantial reduction in the number of tests compared to singular testing. Varying the prevalence from 0%-5% with five possible aliquot numbers suggested the average *halving* percent of test utilized (*PTU*) of 0.30 ± 0.14 compared to singular sample testing, *generalized halving* 0.23 ± 0.09, *splitting* 0.22 ± 0.09, all for *P* = 32 (**Table 1**). In larger initial pool size, *hypercube* required 0.24 ± 0.10 of singular tests, while *indept* required only 0.20 ± 0.10 tests (**Table 1**). Notably, the best savings were seen in cases of the lowest prevalence rates; prevalence rise reduced savings across all methods. The savings were lower in cases of lower number of aliquots, with two aliquots requiring 0.35 ± 0.12, while six aliquots required 0.19 ± 0.08 for *P* = 32 (**Table 1**). The full data set is available in the **Online Supplementary Document**.

**Table 1:** Percent of test that were saved by pooling (*PTS*) across protocol used and number of available aliquots

Protocol and initial pool size (*P*)	Number of aliquots from a single biological sample (swab)					
***t* = 2**	***t* = 3**	***t* = 4**	***t* = 5**	***t* = 6**	**Average, per protocol**
***P* = 32**						
*Halving*	0.54 ± 0.02	0.32 ± 0.04	0.23 ± 0.06	0.20 ± 0.09	0.20 ± 0.09	0.30 ± 0.14
*Generalized halving*	0.29 ± 0.10	0.24 ± 0.09	0.21 ± 0.08	0.20 ± 0.08	0.02 ± 0.09	0.23 ± 0.09
*Splitting*	0.29 ± 0.10	0.22 ± 0.09	0.20 ± 0.09	0.20 ± 0.08	0.20 ± 0.09	0.22 ± 0.09
*Indept*	0.29 ± 0.10	0.21 ± 0.09	0.18 ± 0.08	0.17 ± 0.08	0.17 ± 0.08	0.20 ± 0.09
Average, per number of aliquots	0.35 ± 0.12	0.25 ± 0.09	0.21 ± 0.08	0.19 ± 0.08	0.19 ± 0.08	-
***P* = 64**						
*Hypercube*	0.29 ± 0.10	0.23 ± 0.10	0.23 ± 0.10	0.23 ± 0.10	0.23 ± 0.10	0.24 ± 0.10
*Indept*	0.29 ± 0.10	0.21 ± 0.09	0.18 ± 0.08	0.17 ± 0.08	0.17 ± 0.08	0.20 ± 0.10
Average, per number of aliquots	0.29 ± 0.10	0.22 ± 0.09	0.20 ± 0.09	0.20 ± 0.10	0.20 ± 0.10	-

**Figure 1:**
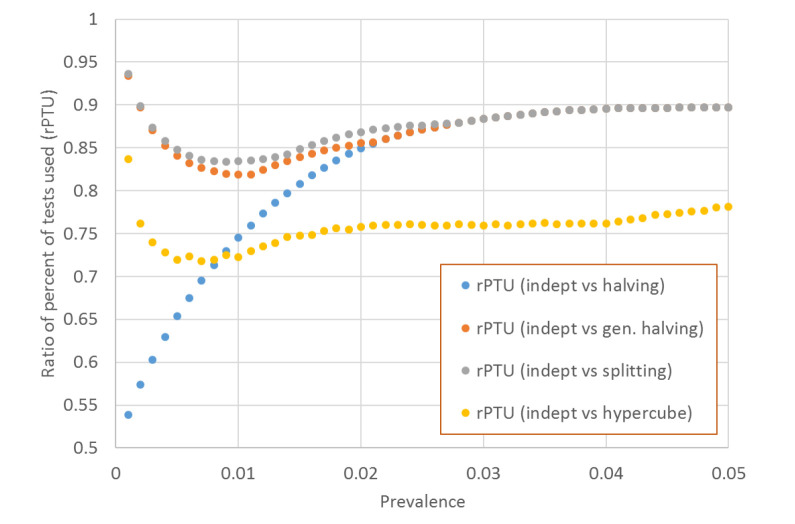
The comparison of ratio of percent of tests used for *indept* protocol and situation of five aliquots (*T* = 5).

The scenario of five aliquots provides the most discriminative power to demonstrate the savings across protocols. The ratio of percentages of tests that were utilized for indept protocol (*rPTU*) with remaining protocols suggested that *indept* outperformed all remaining protocols across a variety of scenarios (**Figure 1**).

We also compared the *hypercube* and *indept* in terms of the time needed to analyse all the samples. For this purpose we developed two separate scenarios, one focusing on time needed to detect the negative samples and the other with time needed to detect positive samples. The initial processing time suggested that *indept* was requiring nearly twice longer processing times at very low prevalence (0%-5%), on average requiring 1.55 ± 0.18 more time for detection of positive results, while it required 1.24 ± 0.14 more time for detection of negative results (Table S10 in the **Online Supplementary Document**). In order to offset this difference, we developed *indeptSp*, a faster version of the protocol, which minimizes the size of terminal pools (**Online Supplementary Document**). Three thresholds were used, corresponding to an average of 10%, 15% and 20% longer processing times. This comes at a certain reduction of savings, but manages to retain savings of about 10%, while having 1.08-1.15 times longer detection times for negative cases and 1.41-1.48 longer times for detection of positive samples, depending on the time extension tolerance (Table S11 in the **Online Supplementary Document**).

## DISCUSSION

The results of this study demonstrate that *indept* protocol had the greatest savings among the analysed pooling protocols. The protocol itself is optimized in a way that it does not depend on the assumed regular structure that some other protocols do, therefore enabling the best adaptability and yields. The problem of longer processing times was surpassed by the development of the *indeptSp*, which minimizes the least informative pools with several samples, which are arbitrarily analysed singularly. This causes certain reduction in total savings, but manages to retain acceptable processing times.

One important issue in comparison of the *indept* with other protocols was the time needed to detect positive vs negative samples. We believe that assigning negative status should have priority over the positive, since anybody referred to testing must be assumed as positive until proven negative. The development of *indeptSp* managed to maintain savings, while minimally extending processing times, a strategy that was deemed acceptable [28]. Notably, this may depend on the epidemic spread, since early stages might focus more on detection of positive cases, their quick isolation and contact tracing efforts.

Given the amount of savings demonstrated for each of the protocols, we claim that any population testing in situation of low prevalence (of mainly asymptomatic subjects) should never be tested by singular testing. This is in line with previous studies [10], and it should probably become a norm in the testing laboratories globally, especially if predicted low levels of seropositivity globally are retained [29].

An extension of the idea of pooling could be to establish computer-based algorithms, which will assist the laboratory staff according to the conditions in which the laboratory operates. Based on the data from previous days or weeks, the software could suggest the pooling tool that would be optimal, including savings, pre-processing time and effort, or other metrics that could assist. This might provide the optimisation that would be capable to provide the best achievable savings, which are direly needed in low resource settings [30].

The limitations of this study include the fact that it was a theoretical development without laboratory validation. In addition, this study assumes no substantial errors in the process, and is therefore an optimistic account of the situation, which might prove less efficient in laboratory conditions, where certain level of errors in testing is expected. Nevertheless, this study provides a theoretical benchmark that could be targeted by the future development and subsequently further adjusted to local conditions. The worldwide demand for diagnostic testing is increasing, making any kind of assistance direly needed. This is why we think that any attempt to do this may serve immensely, especially in low- and middle-income countries, where the cost of human labour is lower, but where the access to testing supplies may be lesser. Next step in the protocol development is the field-testing of the idea, aiming to demonstrate the feasibility of this protocol in real-life surroundings.

## Additional material

Online Supplementary Document
