# Supplementary material for: Optimizing the diagnostic capacity for COVID-19 PCR testing for low resource and high demand settings: The development of information-dependent pooling protocol
Source: J Glob Health. 2020 Dec 30;10(2):020515. doi: 10.7189/jogh.10.020515 (PMC7774501; doi:10.7189/jogh.10.020515)
Supplement: Online Supplementary Document [file jogh-10-020515-s001.zip › Vukicevic_Polasek_supp_m.docx]

**Supplementary material for manuscript *Optimizing the diagnostic capacity for COVID-19 PCR testing for low resource and high demand settings: the development of information-dependent pooling protocol***

Supplementary material Table of contents

[**1. Basic assumptions** 1](#_Toc60048044)

[**2. Halving protocol overview** 2](#_Toc60048045)

[**3. Generalized halving protocol overview** 3](#_Toc60048046)

[**4. Splitting protocol overview** 4](#_Toc60048047)

[**5. Hypercube protocol overview** 6](#_Toc60048048)

[**6. Information dependent protocol (*indept*) overview** 7](#_Toc60048049)

[**Appendix A. Halving protocol** 15](#_Toc60048050)

[**Appendix B. Generalized halving protocol** 17](#_Toc60048051)

[**Appendix C. Splitting protocol** 18](#_Toc60048052)

[**Appendix D. Hypercube protocol** 19](#_Toc60048053)

[**Appendix E. Indept protocol** 20](#_Toc60048054)

[**Appendix F. Expected number of cycles for the information dependent protocol** 21](#_Toc60048055)

[**Appendix G. Development of *indeptSp* protocol** 24](#_Toc60048056)

# **1. Basic assumptions**

The basic assumptions for the method development are:

1. Re-testing of the sample testing produces the same result (for ease of the calculation, errors are omitted at this stage);
2. If at least one sample in the pool yields the positive result when individually tested, the pool would be positive;
3. If all the samples in the pool individually tested give negative results, then the pool would be negative.

Further, we assume that one sample can be tested at most times and that maximal size for the sample pool is. Here we provide the results for and , but all formulas and algorithms can be generalized to any other pool size.

Let us define as the probability that true positive sample is tested positive and let us denote byprobability of false negative. Further, let be the probability that true negative sample is tested as negative and let us denote by probability of false positive. Let be the p of the positive samples in the set of analyzed samples. The probability that test will be positive, which corresponds to a prevalence in a population, is then defined as:

.

# **2. Halving protocol overview**

Suppose that we have a pool of size that is tested positive, and that we want to test each sample. The first protocol under the assessment is the *halving hit pool analysis (HHPA)*:

- If our set () has only one sample and the pool is tested positive, then the (only) sample is positive – no further testing is need.
- If has more than one samples, then we can divide this set in two halves, denoted and . Next, we perform the test on the S1 set. If it tests negative, then all the samples in this set are negative, while at least one sample from must be positive, so *.*
- If is tested positive, then we have no knowledge about, hence besides testing , we should also test . If we test as negative, then all samples in are negative and otherwise.

Suppose that we have a set of samples with no prior knowledge about them. We can then use any set of samples and divide it into batches of size, where is optimized in such a way that expected number of tests per sample is minimal and that and (see Appendix A for details). Optimal values of are presented in the Supplementary Table 1 and the scheme of the protocol is shown in the Supplementary Figure 1.

**Supplementary Table 1.** Initial batch size depending on and for the halving protocol

|  |  | | | | |
| --- | --- | --- | --- | --- | --- |
|  | 2 | 3 | 4 | 5 | 6 |
| 0.1%-2.9% | *2* | *4* | *8* | *16* | *32* |
| 3.0%-5.0% | *2* | *4* | *8* | *16* | *16* |


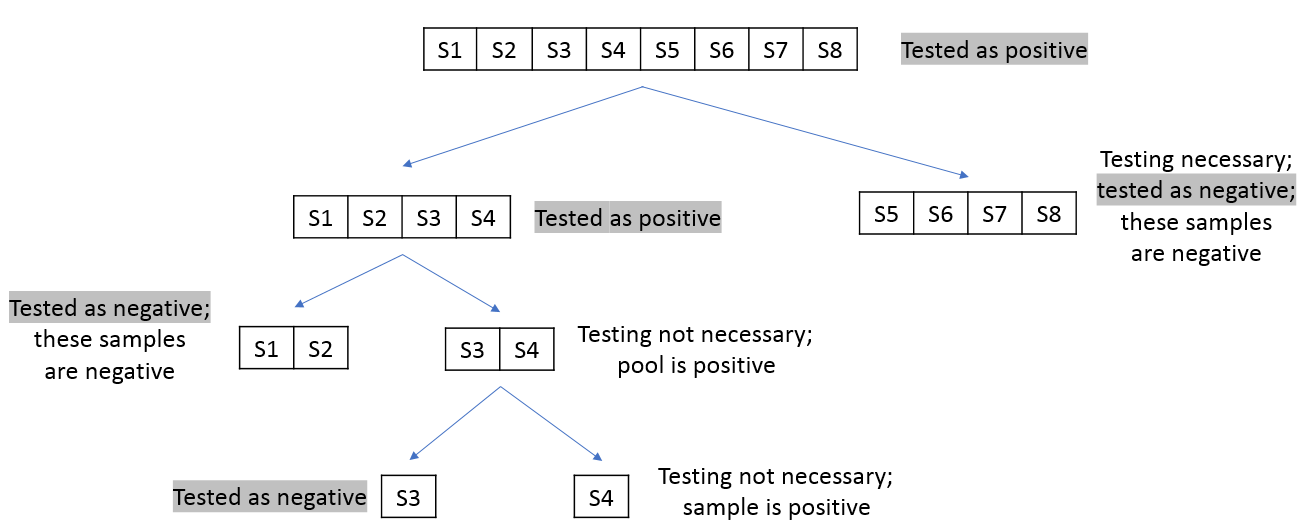
**Supplementary Figure 1**. The halving protocol scheme

# **3. Generalized halving protocol overview**

Suppose that we have a pool of size that is tested positive. We can apply the *generalized* *halving hit pool analysis (GHHPA)* as:

- If has elements, then if , this element is positive and no further testing is need.
- Otherwise, test all elements except the last one. If at least one of them is tested positive, test the last one; and otherwise the last one is positive and it does not need to be tested.
- If has elements where , then this set is divided in two halves and Next, if is tested as negative, then all samples in are negative and must be positive, so . If is tested positive, then we have no knowledge about hence besides testing , we should also test . If we then test as negative, then all samples in are negatives and otherwise .

Finally, any set of samples can be divided into batches of size , where and are optimized in such a way that expected number of tests per sample is minimal (see Appendix B for details) and that: and. Optimal values of are presented in the Supplementary Table 2 and the scheme is shown in the Supplementary Figure 2.

**Supplementary Table 2.** Initial batch size depending on and

|  |  | | | | |
| --- | --- | --- | --- | --- | --- |
|  | 2 | 3 | 4 | 5 | 6 |
| 0.1% | *32* | *16* | *8* | *4* | *2* |
| 0.2% | *23* | *16* | *8* | *4* | *2* |
| 0.3% | *19* | *13* | *8* | *4* | *2* |
| 0.4% | *16* | *11* | *8* | *4* | *2* |
| 0.5% | *15* | *10* | *7* | *4* | *2* |
| 0.6% | *13* | *9* | *7* | *4* | *2* |
| 0.7% | *12* | *9* | *6* | *4* | *2* |
| 0.8% | *12* | *8* | *6* | *4* | *2* |
| 0.9% | *11* | *8* | *5* | *4* | *2* |
| 1.0% | *10* | *7* | *5* | *4* | *2* |
| 1.1%-1.2% | *10* | *7* | *5* | *3* | *2* |
| 1.3% | *9* | *6* | *5* | *3* | *2* |
| 1.4%-1.5% | *9* | *6* | *4* | *3* | *2* |
| 1.6% | *8* | *6* | *4* | *3* | *2* |
| 1.7% | *8* | *6* | *4* | *3* | *2* |
| 1.8%-2.0% | *8* | *5* | *4* | *3* | *2* |
| 2.1% | *7* | *5* | *4* | *3* | *2* |
| 2.2%-2.7% | *7* | *5* | *3* | *2* | *2* |
| 2.8%-2.9% | *6* | *4* | *3* | *2* | *2* |
| 3.0%-3.9% | *6* | *4* | *3* | *2* | *2* |
| 4.0%-4.6% | *5* | *4* | *3* | *2* | *2* |
| 4.7%-5.0% | *5* | *3* | *2* | *2* | *2* |


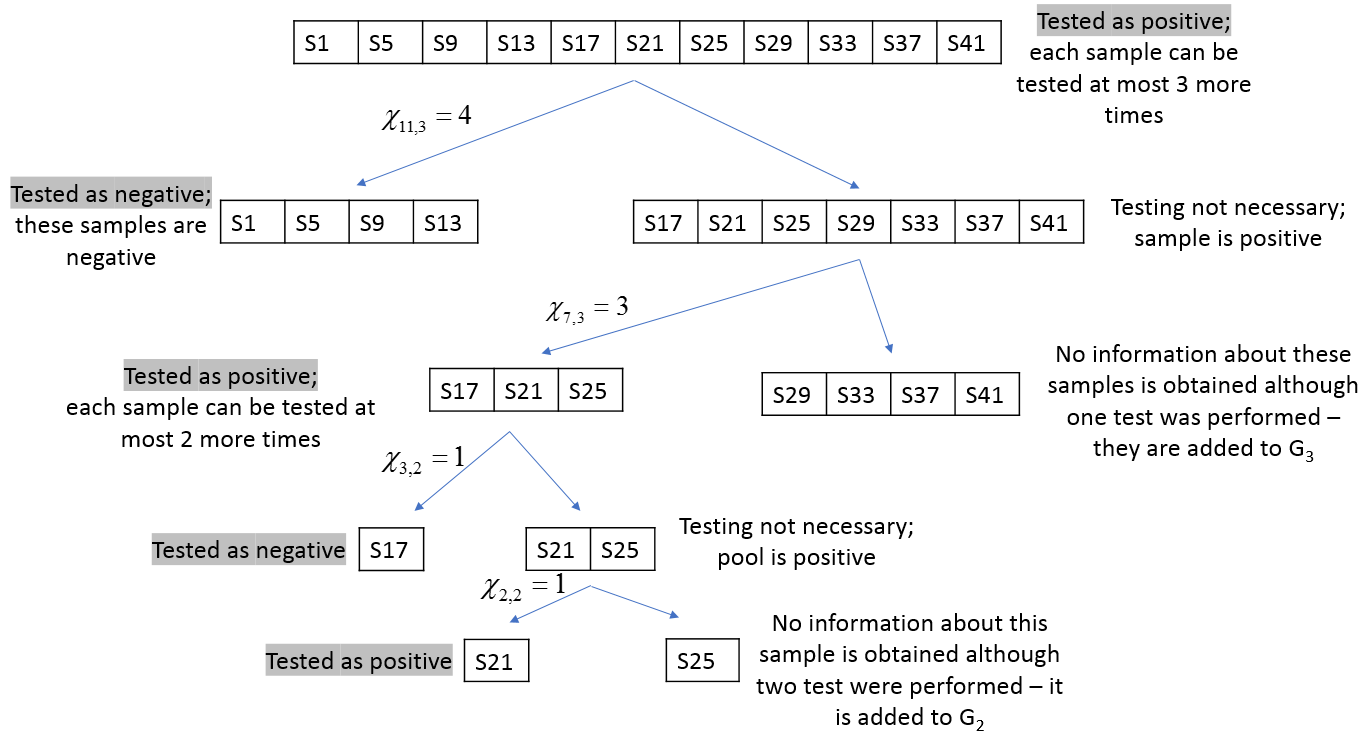


**Supplementary Figure 2**. The generalized halving protocol scheme

# **4. Splitting protocol overview**

Splitting protocol generalizes halving protocol in such a way that one does not necessarily divide the observed set in two sets of equal size, but any number of sets of equal size. Suppose that we have a pool of samples of size , where . The following procedure *splitting hit pool analysis (SHPA)* can be applied:

- If has elements and if , with this element tested as positive, then no further testing is need. Otherwise, test all elements except the last one. If at least one of them is tested as positive, test the last one; and otherwise the last one is positive and it does not need to be tested.
- If has elements, where, then set can be divided into sets of the same sizes. Sets , are tested – if all of them are negative, then is positive and does not need to be tested and otherwise is also tested. For each set that is tested as negative, we know that all samples in it are also negative and for each set that is positive, we apply recursively .

Finally, any set of samples can be divided into batches of size , where factors in this product are optimized in such a way that expected number of tests per sample is minimal (see Appendix C for details) and that: and. Optimal values of the initial batch and every subsequent subdivision are presented in the Supplementary Table 3.

**Supplementary Table 3.** Initial batch size and pool sizes depending on and .

|  |  | | | | |
| --- | --- | --- | --- | --- | --- |
|  | 2 | 3 | 4 | 5 | 6 |
| 0.1% | 32,1 | 32,4,1 | 32,8,2,1 | 32,8,4,2,1 | 32,16,8,4,2,1 |
| 0.2% | 23,1 | 32,4,1 | 32,8,2,1 | 32,8,4,2,1 | 32,16,8,4,2,1 |
| 0.3% | 19,1 | 30,5,1 | 32,8,2,1 | 32,8,4,2,1 | 32,16,8,4,2,1 |
| 0.4% | 16,1 | 30,5,1 | 32,8,2,1 | 32,8,4,2,1 | 32,16,8,4,2,1 |
| 0.5% | 15,1 | 30,5,1 | 32,8,2,1 | 32,8,4,2,1 | 32,16,8,4,2,1 |
| 0.6% | 13,1 | 30,5,1 | 32,8,2,1 | 32,8,4,2,1 | 32,16,8,4,2,1 |
| 0.7% | 12,1 | 30,5,1 | 27,9,3,1 | 32,8,4,2,1 | 32,16,8,4,2,1 |
| 0.8% | 12,1 | 25,5,1 | 27,9,3,1 | 32,8,4,2,1 | 32,16,8,4,2,1 |
| 0.9% | 11,1 | 25,5,1 | 27,9,3,1 | 32,8,4,2,1 | 32,16,8,4,2,1 |
| 1.0%-1.1% | 10,1 | 25,5,1 | 27,9,3,1 | 32,8,4,2,1 | 32,16,8,4,2,1 |
| 1.2% | 10,1 | 20,4,1 | 27,9,3,1 | 32,8,4,2,1 | 32,16,8,4,2,1 |
| 1.3%-1.5% | 9,1 | 20,4,1 | 27,9,3,1 | 32,8,4,2,1 | 32,16,8,4,2,1 |
| 1.6%-2.0% | 8,1 | 16,4,1 | 27,9,3,1 | 24,8,4,2,1 | 32,16,8,4,2,1 |
| 2.1% | 7,1 | 16,4,1 | 27,9,3,1 | 24,8,4,2,1 | 32,16,8,4,2,1 |
| 2.2%-2.3% | 7,1 | 16,4,1 | 18,6,2,1 | 24,8,4,2,1 | 32,16,8,4,2,1 |
| 2.4%-2.7% | 7,1 | 12,3,1 | 18,6,2,1 | 24,8,4,2,1 | 32,16,8,4,2,1 |
| 2.8%-2.9% | 6,1 | 12,3,1 | 18,6,2,1 | 16,8,4,2,1 | 32,16,8,4,2,1 |
| 3.0%-3.2% | 6,1 | 12,3,1 | 18,6,2,1 | 16,8,4,2,1 | 16,8,4,2,1 |
| 3.3%-3.5% | 6,1 | 12,3,1 | 12,4,2,1 | 16,8,4,2,1 | 16,8,4,2,1 |
| 3.6%-3.9% | 6,1 | 9,3,1 | 12,4,2,1 | 16,8,4,2,1 | 16,8,4,2,1 |
| 4.0%-5.0% | 5,1 | 9,3,1 | 12,4,2,1 | 16,8,4,2,1 | 16,8,4,2,1 |


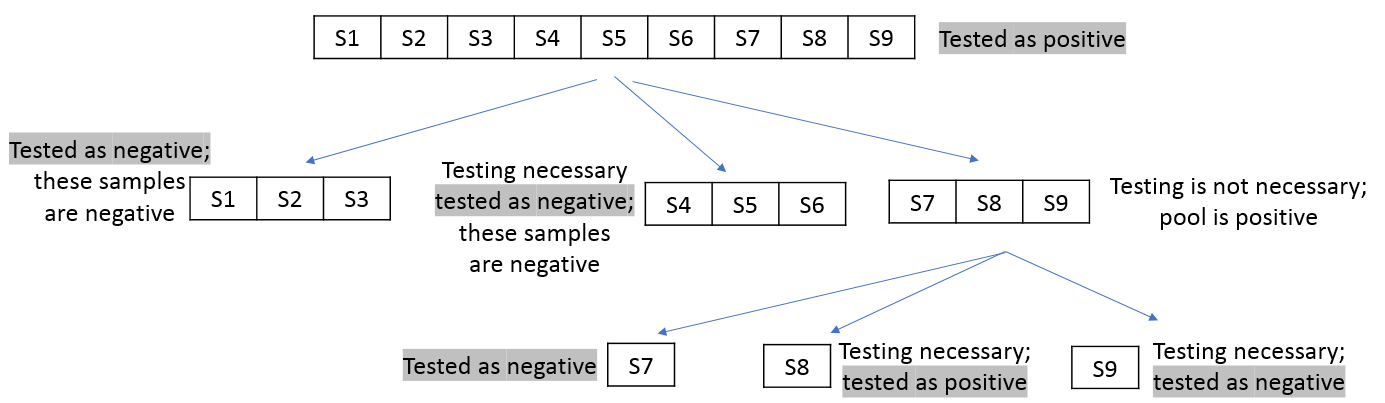


**Supplementary Figure 3**. The splitting protocol scheme

# **5. Hypercube protocol overview**

Let us analyze the expected number of tests of the hypercube-algorithm when each sample is tested at most times, i.e. when – calculation presented here could be easily generalized to arbitrary number of maximal testing per sample, but seems very reasonable for providing comparison between methods considering pool sizes available. Further, we shall limit maximal pool size to . Value is chosen, because it is which allows hypercube method to demonstrate its full potential and makes comparison skewed in the favor of hypercube method. Again, all calculation for can be easily generalized to any other value of .

We shall analyze hypercube starting with initial pools of samples where . Note that the first test is needed for each sample (in the pool of samples). Hence, in the first round expected number of tests per sample is . Sample will be tested for the second time only if at least one sample tested in the same pool with it was positive, i.e. with probability . Hence, the expected number of tests per sample in this round is . Sample will be tested third time only if it was in the second round of testing in the pool of samples in which at least one was positive. Hence, the expected number of tests in this round is . Continuing analogously, we can get that the expected number of tests in the -th round of testing, where is:

.

Hence, expected number of tests per sample is:

.

Therefore, optimal number of tests can be calculated as:

.

Let us denote by and optimal values for given . Note that each negative test will be tested in the first round. In the second round, it will be tested if at least one of in the first pool in which it is tested is positive. It will be tested in the third round if at least one of samples is positive and so on. Hence, the expected number of tests for the negative sample is:

.

# **6. Information dependent protocol (*indept*) overview**

This protocol utilizes all available information from the laboratory work to optimize the savings. In order to do so, the samples classified into sets:

- - set of samples that can be tested more times (or equivalently that are already tested times);
- - families of positive pools, where each positive pool contains samples that can be tested more times.

Obviously, at the beginning of testing, we only have one set and throughout testing every new sample that comes to testing facility is assigned to test . Optimal set of values and that should be tested as a single pool from sets and , respectively, is calculated in the Appendix D. Testing protocol is based on the following procedure:

- In each pool there is only one sample that is tested as positive, so there is no need for testing such pools;
- If , then in each pool test pool of samples. If this pool is negative, then all samples in it are negatives and we know, without testing, that pool of remaining samples is positive, so we add this pool to .
- If this pool is tested as positive, then it is added to set of pools . Note that we have no knowledge about remaining samples except that they can be tested more times, hence we add these samples to the set .
- Test pool of samples from the set . If it is tested as negative, all these samples are negative; and otherwise it is added as new pool to .

Note, that in this algorithm, we try to maximally utilize all information that we have about each sample (Supplementary Figure 4). This will inevitably lead to more complex management of samples, in sense of time and effort required. Optimal values (when maximal pool size is 32) of numbers are presented in the Supplementary Table 4. Optimal values of are all equal to 1 and optimal values the number for are given in Supplementary Tables 5-9.

**Supplementary Table 4.** Optimal values of the numbers

|  |  |  |  |  |  |  |
| --- | --- | --- | --- | --- | --- | --- |
| 0.1% | 1 | 32 | 32 | 32 | 32 | 32 |
| 0.2% | 1 | 23 | 32 | 32 | 32 | 32 |
| 0.3% | 1 | 19 | 32 | 32 | 32 | 32 |
| 0.4% | 1 | 16 | 32 | 32 | 32 | 32 |
| 0.5% | 1 | 15 | 32 | 32 | 32 | 32 |
| 0.6% | 1 | 13 | 32 | 32 | 32 | 32 |
| 0.7% | 1 | 12 | 31 | 32 | 32 | 32 |
| 0.8% | 1 | 12 | 27 | 32 | 32 | 32 |
| 0.9% | 1 | 11 | 26 | 32 | 32 | 32 |
| 1.0% | 1 | 10 | 25 | 32 | 32 | 32 |
| 1.1% | 1 | 10 | 21 | 32 | 32 | 32 |
| 1.3% | 1 | 9 | 21 | 30 | 32 | 32 |
| 1.4% | 1 | 9 | 20 | 28 | 32 | 32 |
| 1.5% | 1 | 9 | 18 | 27 | 32 | 32 |
| 1.6% | 1 | 8 | 17 | 25 | 32 | 32 |
| 1.7% | 1 | 8 | 17 | 25 | 31 | 32 |
| 1.8% | 1 | 8 | 17 | 23 | 29 | 32 |
| 1.9% | 1 | 8 | 16 | 23 | 29 | 32 |
| 2.0% | 1 | 8 | 16 | 22 | 27 | 31 |
| 2.1% | 1 | 7 | 14 | 21 | 25 | 28 |
| 2.2% | 1 | 7 | 14 | 20 | 24 | 27 |
| 2.3% | 1 | 7 | 13 | 19 | 23 | 27 |
| 2.4% | 1 | 7 | 13 | 18 | 23 | 26 |
| 2.5% | 1 | 7 | 13 | 18 | 21 | 24 |
| 2.6% | 1 | 7 | 13 | 17 | 21 | 24 |
| 2.7% | 1 | 7 | 13 | 17 | 20 | 23 |
| 2.8% | 1 | 6 | 12 | 16 | 20 | 22 |
| 2.9% | 1 | 6 | 12 | 16 | 20 | 22 |
| 3.0% | 1 | 6 | 12 | 16 | 20 | 21 |
| 3.1% | 1 | 6 | 11 | 16 | 18 | 21 |
| 3.2% | 1 | 6 | 11 | 16 | 18 | 20 |
| 3.3% | 1 | 6 | 10 | 15 | 18 | 20 |
| 3.4% | 1 | 6 | 10 | 14 | 18 | 19 |
| 3.5% | 1 | 6 | 10 | 14 | 17 | 19 |
| 3.6% | 1 | 6 | 10 | 14 | 17 | 18 |
| 3.7% | 1 | 6 | 10 | 14 | 16 | 18 |
| 3.8% | 1 | 6 | 10 | 13 | 15 | 17 |
| 3.9% | 1 | 6 | 10 | 13 | 15 | 16 |
| 4.0% | 1 | 5 | 10 | 13 | 15 | 16 |
| 4.1% | 1 | 5 | 10 | 13 | 14 | 15 |
| 4.2% | 1 | 5 | 10 | 12 | 14 | 15 |
| 4.3% | 1 | 5 | 10 | 12 | 13 | 14 |
| 4.4% | 1 | 5 | 9 | 11 | 13 | 14 |
| 4.5% | 1 | 5 | 9 | 11 | 13 | 14 |
| 4.6% | 1 | 5 | 9 | 11 | 13 | 14 |
| 4.7% | 1 | 5 | 9 | 11 | 13 | 14 |
| 4.8% | 1 | 5 | 9 | 11 | 13 | 13 |
| 4.9% | 1 | 5 | 9 | 11 | 12 | 13 |
| 5.0% | 1 | 5 | 8 | 11 | 12 | 13 |

**Supplementary Table 5.** Optimal values of the numbers depending on

|  |  |
| --- | --- |
| 2-3 | (0.1%,5.0%)->1 |
| 4-7 | (0.1%,5.0%)->2 |
| 8-13 | (0.1%,5.0%)->3 |
| 14 | (0.1%,1.2%)->4 (1.3%,5.0%)->3 |
| 15 | (0.1%,2.1%)->4 (2.2%,5.0%)->3 |
| 16 | (0.1%,3.2%)->4 (3.3%,5.0%)->3 |
| 17-20 | (0.1%,5.0%)->4 |
| 22 | (0.1%,0.4%)->5 (0.5%,5.0%)->4 |
| 23 | (0.1%,0.7%)->5 (0.8%,5.0%)->4 |
| 24 | (0.1%,1.0%)->5 (1.1%,5.0%)->4 |
| 25 | (0.1%,1.5%)->5 (1.6%,5.0%)->4 |
| 26 | (0.1%,2.8%)->5 (2.9%,5.0%)->4 |
| 27 | (0.1%,2.9%)->5 (3.0%,5.0%)->4 |
| 28-29 | (0.1%,3.1%)->5 (3.2%,5.0%)->4 |
| 30-31 | (0.1%,3.9%)->5 (4.0%,5.0%)->4 |
| 32 | (0.1%,0.2%)->6 (0.3%,4.1%)->5 (4.2%,5.0%)->4 |

**Supplementary Table 6.** Optimal values of the numbers depending on

|  |  |
| --- | --- |
| 2-3 | (0.1%,5.0%)->1 |
| 4-6 | (0.1%,5.0%)->2 |
| 7-8 | (0.1%,5.0%)->3 |
| 9 | (0.1%,0.2%)->4 (0.3%,5.0%)->3 |
| 10 | (0.1%,2.4%)->4 (2.5%,5.0%)->3 |
| 11 | (0.1%,5.0%)->4 |
| 12 | (0.1%,4.7%)->5 (4.8%,5.0%)->4 |
| 13-16 | (0.1%,5.0%)->5 |
| 17 | (0.1%,0.9%)->6 (1.0%,5.0%)->5 |
| 18 | (0.1%,3.3%)->6 (3.4%,5.0%)->5 |
| 19 | (0.1%,2.0%)->7 (2.1%,3.8%)->6 (3.9%,5.0%)->5 |
| 20 | (0.1%,2.4%)->7 (2.5%,4.5%)->6 (4.6%,5.0%)->5 |
| 21 | (0.1%,2.9%)->7 (3.0%,4.8%)->6 (4.9%,5.0%)->5 |
| 22 | (0.1%,2.9%)->7 (3.0%,5.0%)->6 |
| 23 | (0.1%,4.1%)->7 (4.2%,5.0%)->6 |
| 24 | (0.1%,4.3%)->7 (4.4%,5.0%)->6 |
| 25 | (0.1%,0.2%)->8 (0.3%,4.7%)->7 (4.8%,5.0%)->6 |
| 26 | (0.1%,1.4%)->8 (1.5%,5.0%)->7 |
| 27 | (0.1%,1.8%)->8 (1.9%,5.0%)->7 |
| 28 | (0.1%,1.3%)->9 (1.4%,2.0%)->8 (2.1%,5.0%)->7 |
| 29 | (0.1%,0.8%)->10 (0.9%,1.5%)->9 (1.6%,2.2%)->8 (2.3%,5.0%)->7 |
| 30 | (0.1%,1.0%)->10 (1.1%,1.7%)->9 (1.8%,2.3%)->8 (2.4%,5.0%)->7 |
| 31 | (0.1%,1.1%)->10 (1.2%,1.7%)->9 (1.8%,2.8%)->8 (2.9%,5.0%)->7 |
| 32 | (0.1%,1.1%)->10 (1.2%,2.2%)->9 (2.3%,3.1%)->8 (3.2%,5.0%)->7 |

**Supplementary Table 7.** Optimal values of the numbers depending on

|  |  |
| --- | --- |
| 2-3 | (0.1%,5.0%)->1 |
| 4-6 | (0.1%,5.0%)->2 |
| 7 | (0.1%,5.0%)->3 |
| 8-11 | (0.1%,5.0%)->4 |
| 12 | (0.1%,2.6%)->5 (2.7%,5.0%)->4 |
| 13 | (0.1%,5.0%)->5 |
| 14 | (0.1%,4.7%)->6 (4.8%,5.0%)->5 |
| 15 | (0.1%,3.3%)->7 (3.4%,5.0%)->6 |
| 16 | (0.1%,3.7%)->7 (3.8%,5.0%)->6 |
| 17 | (0.1%,4.2%)->7 (4.3%,5.0%)->6 |
| 18-19 | (0.1%,5.0%)->7 |
| 20 | (0.1%,0.3%)->8 (0.4%,5.0%)->7 |
| 21 | (0.1%,0.6%)->8 (0.7%,5.0%)->7 |
| 22 | (0.1%,3.0%)->8 (3.1%,5.0%)->7 |
| 23 | (0.1%,0.6%)->9 (0.7%,3.2%)->8 (3.3%,5.0%)->7 |
| 24 | (0.1%,0.2%)->10 (0.3%,2.4%)->9 (2.5%,3.5%)->8 (3.6%,5.0%)->7 |
| 25 | (0.1%,1.9%)->10 (2.0%,2.6%)->9 (2.7%,3.9%)->8 (4.0%,5.0%)->7 |
| 26 | (0.1%,1.8%)->11 (1.9%,2.0%)->10 (2.1%,2.9%)->9 (3.0%,4.5%)->8 (4.6%,5.0%)->7 |
| 27 | (0.1%,1.1%)->12 (1.2%,1.9%)->11 (2.0%,2.2%)->10 (2.3%,3.4%)->9 (3.5%,4.7%)->8 (4.8%,5.0%)->7 |
| 28 | (0.1%,1.2%)->12 (1.3%,2.1%)->11 (2.2%,2.6%)->10 (2.7%,3.5%)->9 (3.6%,5.0%)->8 |
| 29 | (0.1%,1.3%)->12 (1.4%,2.5%)->11 (2.6%,4.2%)->9 (4.3%,5.0%)->8 |
| 30 | (0.1%,1.5%)->12 (1.6%,2.5%)->11 (2.6%,3.1%)->10 (3.2%,4.3%)->9 (4.4%,5.0%)->8 |
| 31 | (0.1%,1.5%)->12 (1.6%,2.9%)->11 (3.0%,3.5%)->10 (3.6%,4.5%)->9 (4.6%,5.0%)->8 |
| 32 | (0.1%,1.8%)->12 (1.9%,3.3%)->11 (3.4%,3.7%)->10 (3.8%,4.7%)->9 (4.8%,5.0%)->8 |

**Supplementary Table 8.** Optimal values of the numbers depending on

|  |  |
| --- | --- |
| 2-3 | (0.1%,5.0%)->1 |
| 4-6 | (0.1%,5.0%)->2 |
| 7 | (0.1%,5.0%)->3 |
| 8-12 | (0.1%,5.0%)->4 |
| 13 | (0.1%,5.0%)->5 |
| 14 | (0.1%,5.0%)->6 |
| 15 | (0.1%,4.6%)->7 (4.7%,5.0%)->6 |
| 16 | (0.1%,3.3%)->8 (3.4%,5.0%)->7 |
| 17 | (0.1%,3.6%)->8 (3.7%,5.0%)->7 |
| 18 | (0.1%,3.9%)->8 (4.0%,5.0%)->7 |
| 19 | (0.1%,4.4%)->8 (4.5%,5.0%)->7 |
| 20 | (0.1%,4.8%)->8 |
| 21-23 | (0.1%,5.0%)->8 |
| 24 | (0.1%,3.7%)->9 (3.8%,5.0%)->8 |
| 25 | (0.1%,2.5%)->10 (2.6%,4.0%)->9 (4.1%,5.0%)->8 |
| 26 | (0.1%,1.1%)->11 (1.2%,3.1%)->10 (3.2%,4.3%)->9 (4.4%,5.0%)->8 |
| 27 | (0.1%,0.4%)->12 (0.5%,2.7%)->11 (2.8%,3.3%)->10 (3.4%,4.7%)->9 (4.8%,5.0%)->8 |
| 28 | (0.1%,2.1%)->12 (2.2%,2.9%)->11 (3.0%,3.6%)->10 (3.7%,5.0%)->9 |
| 29 | (0.1%,2.0%)->13 (2.1%,2.2%)->12 (2.3%,3.0%)->11 (3.1%,3.9%)->10 (4.0%,5.0%)->9 |
| 30 | (0.1%,1.6%)->14 (1.7%,2.1%)->13 (2.2%,2.3%)->12 (2.4%,3.3%)->11 (3.4%,4.2%)->10 (4.3%,5.0%)->9 |
| 31 | (0.1%,1.3%)->15 (1.4%,1.7%)->14 (1.8%,2.3%)->13 (2.4%,2.5%)->12 (2.6%,3.5%)->11 (3.6%,4.6%)->10 (4.7%,5.0%)->9 |
| 32 | (0.1%,1.3%)->15 (1.4%,1.8%)->14 (1.9%,2.5%)->13 (2.6%,3.8%)->11 (3.9%,4.9%)->10 (5.0%,5.0%)->9 |

**Supplementary Table 9.** Optimal values of the numbers depending on

|  |  |
| --- | --- |
| 2-3 | (0.1%,5.0%)->1 |
| 4-6 | (0.1%,5.0%)->2 |
| 7 | (0.1%,5.0%)->3 |
| 8-12 | (0.1%,5.0%)->4 |
| 13 | (0.1%,5.0%)->5 |
| 14 | (0.1%,5.0%)->6 |
| 15 | (0.1%,5.0%)->7 |
| 16 | (0.1%,4.2%)->8 (4.3%,5.0%)->7 |
| 17 | (0.1%,4.5%)->8 (4.6%,5.0%)->7 |
| 18 | (0.1%,4.9%)->8 (5.0%,5.0%)->7 |
| 19-23 | (0.1%,5.0%)->8 |
| 24 | (0.1%,2.6%)->9 (2.7%,5.0%)->8 |
| 25 | (0.1%,4.0%)->9 (4.1%,5.0%)->8 |
| 26 | (0.1%,3.5%)->10 (3.6%,4.3%)->9 (4.4%,5.0%)->8 |
| 27 | (0.1%,3.1%)->11 (3.2%,3.7%)->10 (3.8%,4.6%)->9 (4.7%,5.0%)->8 |
| 28 | (0.1%,2.7%)->12 (2.8%,3.3%)->11 (3.4%,3.9%)->10 (4.0%,4.9%)->9 (5.0%,5.0%)->8 |
| 29 | (0.1%,2.5%)->13 (2.6%,2.8%)->12 (2.9%,3.4%)->11 (3.5%,4.2%)->10 (4.3%,5.0%)->9 |
| 30 | (0.1%,2.2%)->14 (2.3%,2.6%)->13 (2.7%,2.9%)->12 (3.0%,3.7%)->11 (3.8%,4.5%)->10 (4.6%,5.0%)->9 |
| 31 | (0.1%,1.9%)->15 (2.0%,2.3%)->14 (2.4%,2.7%)->13 (2.8%,3.1%)->12 (3.2%,3.9%)->11 (4.0%,4.9%)->10 (5.0%,5.0%)->9 |
| 32 | (0.1%,1.5%)->16 (1.6%,2.0%)->15 (2.1%,2.4%)->14 (2.5%,2.9%)->13 (3.0%,3.3%)->12 (3.4%,4.2%)->11 (4.3%,5.0%)->10 |

**Supplementary Figure 4**. The information dependent protocol scheme. Example of the application of the information protocol for and . State of laboratory at the begging of one round of testing is presented in a) – samples that will be involved in the next round of testing are emphasized by gray color (, , and ) . That round of testing is presented in b1-b4) where b1) explains testing of samples that have only one more test allowed, b2) two tests allowed, b3) three tests allowed and b4) four tests allowed. State of laboratory after that round of testing finished is presented in c) – samples involved in this round that are returned for further analyses are emphasized by gray color.

a)


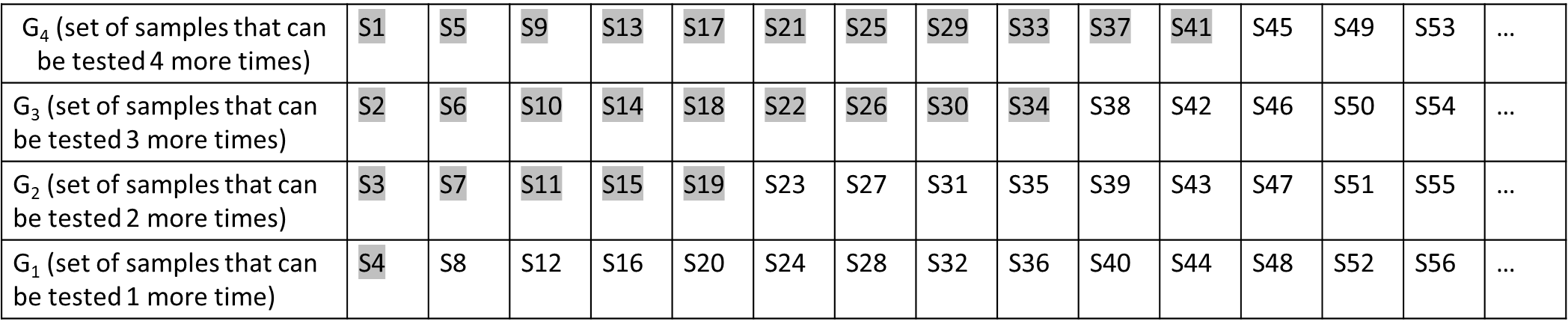


b1)


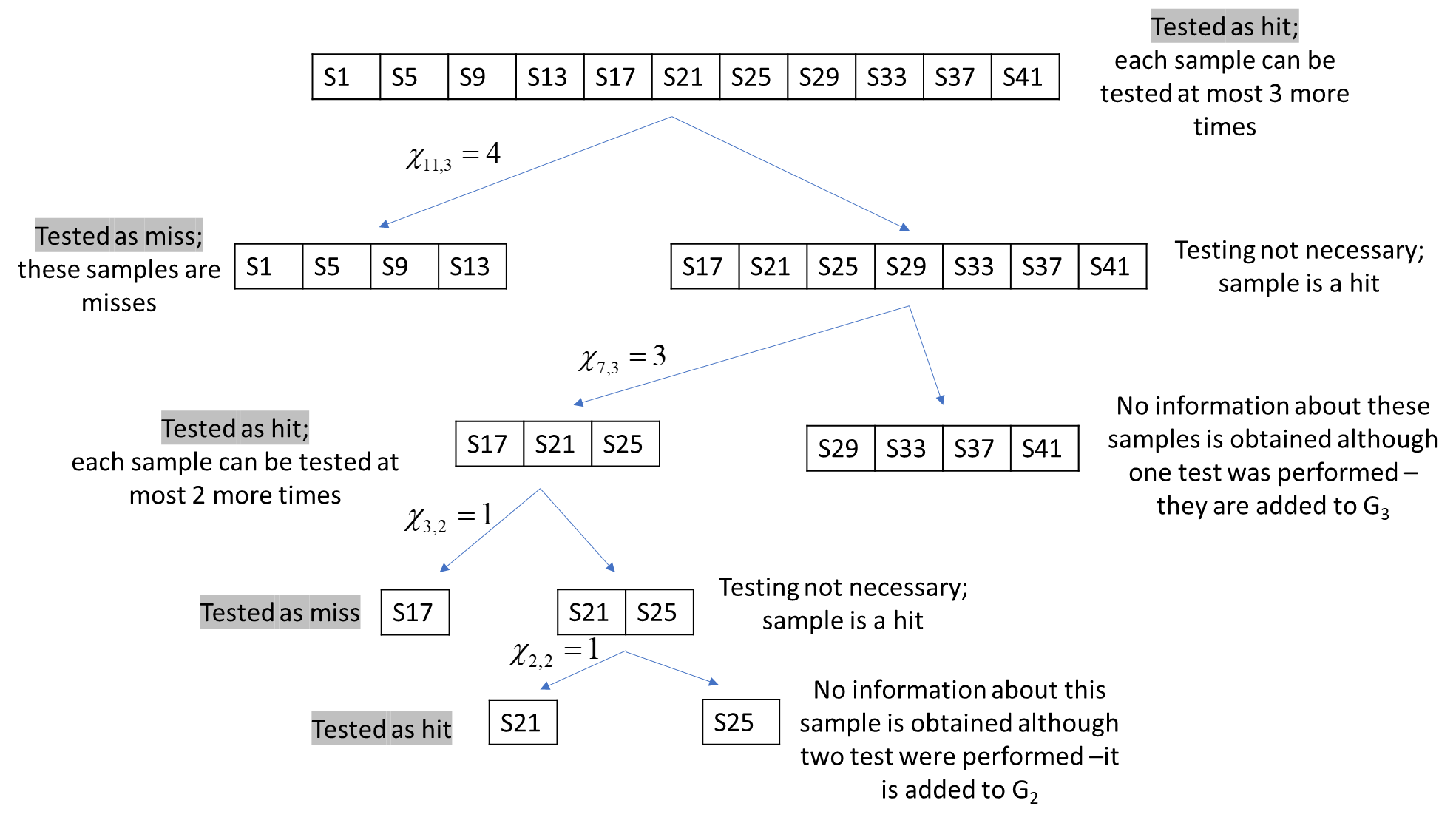


b2)


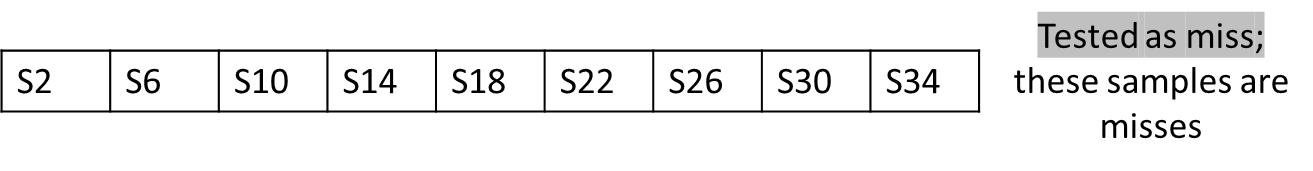


b3)


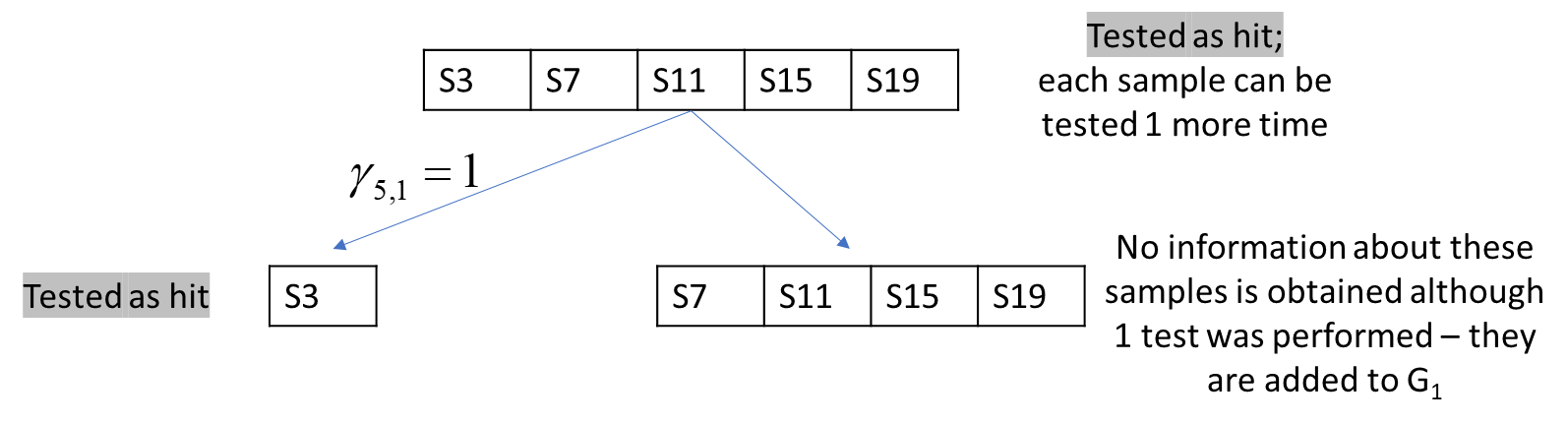


b4)


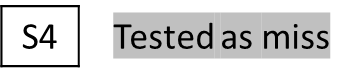


c)


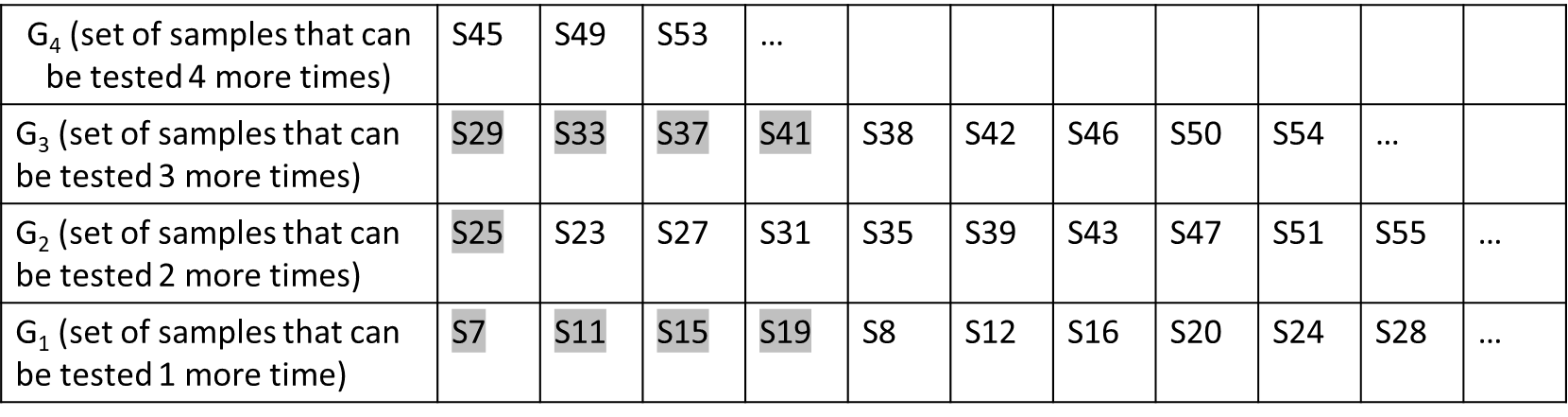


One problem that we noted is the longer duration of the processing of the entire pool for *indept*, compared to other protocols (Supplementary Table 5).

**Supplementary Table 10**. Comparison of the number of cycles needed to complete the entire diagnostic process for pool size *P*=64, when *hypercube* and *indept* protocol are compared

| Prevalence | Number of cycles needed, positive result | | Ratio | Number of cycles needed, negative result | | Ratio |
| --- | --- | --- | --- | --- | --- | --- |
|  | *hypercube* | *indept* |  | *hypercube* | *indept* |  |
| 0.2 | 4 | 7.62 | 1.91 | 1 | 1.15 | 1.18 |
| 0.4 | 4 | 7.65 | 1.91 | 2 | 1.29 | 1.32 |
| 0.6 | 4 | 7.29 | 1.82 | 2 | 1.42 | 1.29 |
| 0.8 | 4 | 7.00 | 1.75 | 2 | 1.53 | 1.23 |
| 1 | 4 | 6.68 | 1.67 | 2 | 1.64 | 1.16 |
| 1.2 | 4 | 6.55 | 1.64 | 2 | 1.39 | 1.40 |
| 1.4 | 4 | 6.20 | 1.55 | 2 | 1.44 | 1.33 |
| 1.6 | 4 | 6.15 | 1.54 | 2 | 1.50 | 1.28 |
| 1.8 | 4 | 6.04 | 1.51 | 2 | 1.55 | 1.26 |
| 2 | 4 | 5.93 | 1.48 | 2 | 1.60 | 1.24 |
| 2.2 | 4 | 5.76 | 1.44 | 2 | 1.65 | 1.18 |
| 2.4 | 4 | 5.58 | 1.39 | 2 | 1.69 | 1.13 |
| 2.6 | 4 | 5.56 | 1.39 | 2 | 1.74 | 1.12 |
| 2.8 | 4 | 5.44 | 1.36 | 2 | 1.78 | 1.09 |
| 3 | 4 | 5.45 | 1.36 | 2 | 1.82 | 1.10 |
| 3.2 | 4 | 5.43 | 1.36 | 2 | 1.86 | 1.10 |
| 3.4 | 4 | 5.26 | 1.31 | 2 | 1.90 | 1.03 |
| 3.6 | 4 | 5.27 | 1.32 | 2 | 1.94 | 1.04 |
| 3.8 | 4 | 5.14 | 1.28 | 2 | 1.98 | 1.00 |
| 4 | 3 | 5.15 | 1.72 | 2 | 1.36 | 1.49 |
| 4.2 | 3 | 4.99 | 1.66 | 2 | 1.37 | 1.44 |
| 4.4 | 3 | 4.85 | 1.62 | 2 | 1.39 | 1.39 |
| 4.6 | 3 | 4.86 | 1.62 | 2 | 1.40 | 1.40 |
| 4.8 | 3 | 4.87 | 1.62 | 2 | 1.42 | 1.41 |
| 5 | 3 | 4.85 | 1.62 | 2 | 1.43 | 1.41 |
| Average±SD |  |  | 1.55±0.18 |  |  | 1.24±0.14 |

In order to offset this, we modified the original *indept* protocol and enabled the initial branching, which optimizes the protocol for maximum savings (the original *indept*) or for maximum speed (*indeptSp*). The development of this protocol was made under the assumption that the negative test result is a priority, since the suspect cases (those who are sent to be tested), must be assumed as positive until they get the negative result (if truly negative). The main change in *indeptSp* compared to the *indept* is that at the last *T* cycle, all samples from the pool undergo individual testing. This reduces the waiting times, but also increases the number of tests that are required to complete the pooling and diagnostics process. In order to optimize the *indeptSp* protocol, we arbitrarily set the delay at 10%, 15% or 20% longer than the *hypercube*, and measured the gains in number of tests reduction. The results had shown that *indeptSp* outperformed the *hypercube* in all instances, and provided marginally longer processing times than *hypercube* (Supplementary Table 6).

**Supplementary Table 11**. Comparison of *hypercube* and *indeptSp* protocol outcomes by the ratio of percent of test utilized (*rPTU*), depending on the prevalence rates in the population, with three initial time delay settings of 10, 15 or 20% longer processing times

| Approach | Parameter | Values |
| --- | --- | --- |
| Processing time extension of 10% | Ratio of tests used (indept over hypercube) | 0.84±0.04 |
|  | Waiting, time negative cases | 1.08±0.03 |
|  | Waiting, time positive cases | 1.41±0.15 |
| Processing time extension of 15% | Ratio of tests used (indept over hypercube) | 0.82±0.02 |
|  | Waiting, time negative cases | 1.12±0.04 |
|  | Waiting, time positive cases | 1.45±0.15 |
| Processing time extension of 20% | Ratio of tests used (indept over hypercube) | 0.82±0.02 |
|  | Waiting, time negative cases | 1.15±0.06 |
|  | Waiting, time positive cases | 1.48±0.17 |

# **Appendix A. Halving protocol**

Let us inductively calculate the expected number of tests needed to analyze samples in the pool of size , which is positive. If , then there is only one sample in the pool and that sample is positive. Hence, no testing is needed, i.e. .

Now, let us assume that and let and be two halves of the observed pool. Let us distinguish three possibilities (that tester does not know in advance):

CASE 1: is negative.

Probability of this case is:

After testing , tester knows that is positive, hence the required number of tests is:

.

CASE 2: is positive and is negative.

Probability of this case is:

and required number of tests is:

.

CASE 3: and are both positive:

Probability of this case is:

and required number of tests is:

.

Summing up, we get:

The required number of tests needed by this protocol if we start by testing pool of size about which we do now know anything is:

Finally, is selected such that it minimizes while respecting the conditions on the maximal pool size and maximal number of tests per sample, i.e. and .

# **Appendix B. Generalized halving protocol**

Let us inductively calculate the expected number of tests needed to analyze samples in the pool of size , which was tested as positive. Let us observe the case . If , then we test first samples and we test the last sample unless the first tests are negative. Probability of such event is:

,

Hence,

for . It can be seen that the above formula is also true for , because . Now, let us assume that and let and be two halves of the observed pool. Let us distinguish three possibilities (that tester does not know in advance) and apply calculation very similar to one presented in the Appendix A.

CASE 1: is negative.

Probability of this case is:

After testing , tester knows that is positive, hence the required number of tests is:

.

CASE 2: is positive and is negative.

Probability of this case is:

and required number of tests is:

.

CASE 3: and are both positive:

Probability of this case is:

and required number of tests is:

.

Summing up, we get:

The required number of tests needed by this protocol if we start by testing pool of size about which we do now know anything is:

Finally, and are selected such that they minimizes while respecting the conditions on the maximal pool size and maximal number of tests per sample, i.e. that and .

# **Appendix C. Splitting protocol**

Let us inductively calculate the expected number of tests needed to analyze samples in the pool of size , which was tested as positive. The case is solved completely analogously as inductive base in the Appendix B and we get:

.

Now, let us assume that and let ,…, be division of the observed pool in sub-pools having the same number of elements. Note that probability that all sets will be negative is:

.

Hence, expected initial number of tests to determine which of the pools are positives:

.

Probability that any set is positive is:

.

Hence, expected number of positive results is:

.

Therefore,

The required number of tests needed by this protocol if we start by testing pool of size about which we do now know anything is:

Finally, are selected such that they minimizes while respecting the conditions on the maximal pool size and maximal number of tests per sample, i.e. that and .

# **Appendix D. Hypercube protocol**

Let us analyze the expected number of tests of the hypercube-algorithm when each sample is tested at most times, i.e. when – calculation presented here could be easily generalized to arbitrary number of maximal testing per sample, but seems very reasonable for providing comparison between methods considering pool sizes available. Further, we shall limit maximal pool size to . Value is chosen, because it is which allows hypercube method to demonstrate its full potential savings. Notably, this value can be easily generalized to any other value of .

We shall analyze hypercube protocol by starting with initial pools of samples where. Note that the first test is needed for each sample (in the pool of samples). Hence, in the first testing cycle expected number of tests per sample is . Sample will be tested for the second time only if at least one sample tested in the same pool with it was positive, i.e. with probability. Hence, the expected number of tests per sample in this cycle is . Sample will be tested third time only if it was in the second cycle of testing in the pool of samples in which at least one was positive. Hence, the expected number of tests in this cycle is . Continuing analogously, we can get that the expected number of tests in the -th cycle of testing, where is:

.

Hence, expected number of tests per sample is:

.

Therefore, optimal number of tests can be calculated as:

.

Let us denote by and optimal values for given . Note that each negative test will be tested in the first cycle. In the second cycle, it will be tested if at least one of in the first pool in which it is tested is positive. It will be tested in the third cycle if at least one of samples is positive and so on. Hence, the expected number of tests for the negative sample is:

.

# **Appendix E. Indept protocol**

Let us calculate all values and by induction on . It can be easily seen that . Note that if we need to analyze hit pool of samples on each of which only on test is allowed, the only possibility is to test all of them individually except possibly the last one (if all other results are misses, then the last one is hit). Hence,

.

Now, let us solve the problem for . First, let us calculate . Suppose that we test samples about which nothing is known. Probability that this pool is a miss is and probability that it is a hit is , hence, expected number of tests needed to examine this samples is:

.

Optimizing in order to minimize expected number of tests per sample, we get:

, for each .

Now, let us calculate by induction on . If , then the only sample in the pool is hit, hence. Let us assume that . Note that probability that pool of size , contained in the hit pool of size , is a miss is equal to:

.

In that case, we know that all these samples are misses and the remaining samples create hit pool, so expected number of tests needed to test them is .

On the other, hand if pool of size is hit, then we have no knowledge about remaining samples, hence:

This gives us all necessary formulas for the calculation of and .

# **Appendix F. Expected number of cycles for the information dependent protocol**

Let us calculate the expected number of cycles needed for the sample to be processed in the information dependent protocol. Let denote the expected number of sample-cycles (unit analogous to man-hours for different job tasks, e.g. if 3 samples are processed in 2 cycles and 1 sample in 3 cycle, we have 9 sample-cycles) for all samples in the positive pool of samples that can be tested at most more times and let denotes expected waiting time per sample among the set of all samples that can be tested at most times (measured in cycles). Let and denote the same concepts when we restrict our attention to positive samples and let and denote the same concepts when we restrict our attention to negative samples.

Note that

because all the samples except the last one are tested in the first cycles and the conclusion about the last one cannot be made if one of the other samples is a positive, hence in that case it must be tested in the next cycle.

It holds that:

because all samples are tested ones. If the result is negative – all samples are misses and otherwise they form a positive pool of samples that can be tested at most times.

Further,

because after testing sub-pool of samples - negative result implies that all these samples are negative and remaining samples create positive pool of samples that can be tested at most more times; positive result implies that we have a positive result in pool of samples that can be tested at most times and samples that we do not know anything about that can be tested at most more times.

Let us note that conditional probability that sample is negative in the positive pool of size is equal to

.

Hence, conditional probability that sample is positive in the positive pool of size is equal to:

.

Now, let us restrict out attention to positive samples. The calculation holds analogously as above:

It holds that:

and that:

Finally, let us restrict our attention to negative samples:

It holds that:

;

The calculation can be checked by testing it as:

.


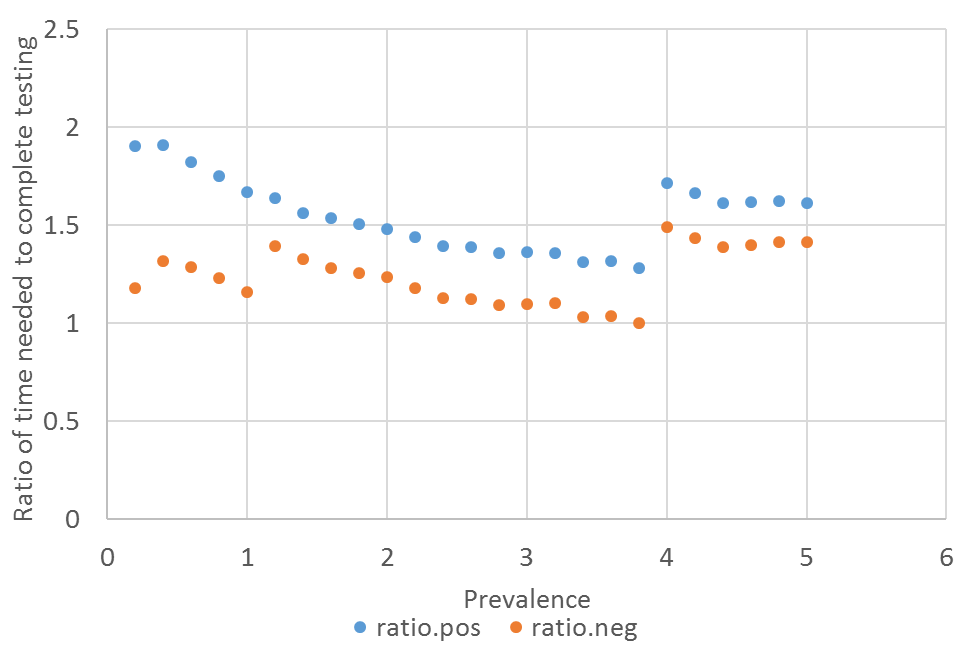


**Supplementary Figure 5**. Comparison of processing time for hypercube and indept protocols, in detection of positive and negative results

# **Appendix G. Development of *indeptSp* protocol**

This version of *indept* protocol utilizes previous assumptions - only in selecting value of it does not simply minimizes the number of tests, but minimizes this number under assumption that the expected time for processing negative sample may be at most 110% (respectively 115% and 120%) of expected time needed for processing negative sample by hypercube algorithm. This may lead to selection of the smaller values of (Supplementary Table 7).

**Supplementary Table 12.** Values of for indeptSp when maximal expected time for processing negative sample may be at most 110% (respectively 115% and 120%) of expected time needed for processing negative sample by hypercube algorithm

|  | Negative sample processing time delay | | |
| --- | --- | --- | --- |
|  | 110% | 115% | 120% |
| 0.2% | 47 | 59 | 64 |
| 0.4% | 40 | 45 | 50 |
| 0.6% | 36 | 41 | 45 |
| 0.8% | 34 | 38 | 42 |
| 1.0% | 32 | 36 | 37 |
| 1.2% | 19 | 21 | 23 |
| 1.4% | 18 | 20 | 22 |
| 1.6% | 18 | 20 | 21 |
| 1.8% | 17 | 19 | 21 |
| 2.0% | 16 | 18 | 20 |
| 2.2% | 16 | 18 | 20 |
| 2.4% | 16 | 18 | 18 |
| 2.6% | 16 | 17 | 17 |
| 2.8% | 16 | 16 | 16 |
| 3.0% | 15 | 16 | 16 |
| 3.2% | 15 | 16 | 16 |
| 3.4% | 14 | 14 | 14 |
| 3.6% | 14 | 14 | 14 |
| 3.8% | 13 | 13 | 13 |
| 4.0% | 6 | 7 | 8 |
| 4.2% | 6 | 7 | 7 |
| 4.4% | 6 | 7 | 7 |
| 4.6% | 6 | 7 | 7 |
| 4.8% | 6 | 7 | 7 |
| 5.0% | 6 | 7 | 7 |
